# Dysfunction of cerebrospinal fluid circulation and neurovascular decoupling in end-stage renal disease patients with mild cognitive impairment

**DOI:** 10.1007/s11357-025-01901-5

**Published:** 2025-10-12

**Authors:** Zhaoyao Luo, Lurui Bo, Xinyi Zhu, Qiange Zhu, Huijie Yuan, Wen Gu, Peng Li, Yihao Peng, Xiang Chen, Ming Zhang, Shaohui Ma, Junya Mu

**Affiliations:** 1 Department of Medical Imaging, the First Affiliated Hospital of Xi’an Jiaotong University, Xi’an, 710061 Shaanxi Province China; 2 Xi’an Jiaotong University, Xi’an, 710061 Shaanxi Province China; 3 Department of Radiology, the Second Hospital of the Air Force Medical University, Xi’an, Shaanxi Province China; 4 Department of Medical Imaging, Nuclear 215 Hospital of Shaanxi Province, Xianyang, Shaanxi Province China; 5 The Second Affiliated Hospital, Hengyang Medical School, University of South China, Hengyang, 421001 Hunan China

**Keywords:** End-stage renal disease, Mild cognitive impairment, Cerebrospinal fluid circulation, Resting-state functional magnetic resonance imaging, Neurovascular coupling, Diffusion tensor imaging

## Abstract

End-stage renal disease (ESRD) patients frequently experience mild cognitive impairment (MCI), which significantly affects their quality of life. Although dysfunctions in cerebrospinal fluid (CSF) circulation and neurovascular coupling (NVC) have been implicated in cognitive impairment, their interrelationship remains unclear. This study aimed to investigate the association between CSF circulation dysfunction and NVC disruption in ESRD patients with MCI. We enrolled 68 ESRD patients with MCI and 65 healthy controls (HCs). CSF circulation was assessed using choroid plexus volume (CPV), the diffusion tensor imaging along the perivascular space (DTI-ALPS) index, and perivascular space volume fraction (PVSVF). NVC was evaluated using coupling metrics between cerebral blood flow (CBF) and resting-state functional magnetic resonance imaging (MRI) indicators: amplitude of low-frequency fluctuation (ALFF), fractional ALFF (fALFF), and regional homogeneity (ReHo). Cognitive performance was measured using a battery of neuropsychological tests. Compared to HCs, ESRD patients showed significantly increased CPV (*P* < 0.001) and enlarged PVSVF (*P* < 0.05), along with a decreased DTI-ALPS index (*P* < 0.01). Global and regional NVC metrics were significantly reduced in ESRD patients (*P* < 0.001). Moreover, regional NVC metrics were negatively correlated with CPV. Mediation analysis further revealed that CBF-ReHo coupling in the left cingulate gyrus pregenual area 32 mediated the relationship between elevated LCPV and cognitive decline. These findings suggest that CSF circulation dysfunction and impaired NVC are interrelated in ESRD-MCI. Disruption of CSF circulation may contribute to cognitive decline by impairing NVC, highlighting both as potential targets for early diagnosis and intervention in ESRD-related cognitive impairment.

**Supplementary Information:**

The online version contains supplementary material available at 10.1007/s11357-025-01901-5.

## Introduction

End-stage renal disease (ESRD) represents the final stage of chronic kidney disease (CKD), where kidney function is permanently reduced to less than 10% of its normal capacity [1]. Mild cognitive impairment (MCI) affects 16–38% of ESRD patients, with 5–10% progressing to dementia—a trajectory characterized by accelerated cognitive decline and reduced quality of life [2, 3]. Previous studies suggest that both cerebrospinal fluid (CSF) circulation dysfunction and impaired neurovascular coupling (NVC) contribute to cognitive impairment in ESRD patients [4, 5], yet their interplay in cognitive impairment remains underexplored. Thus, investigating the biological mechanisms and biomarkers of MCI in ESRD, particularly from the perspective of CSF circulation and NVC dysfunction, is crucial for early diagnosis and intervention in ESRD patients with MCI.

A key pathway involved in waste clearance is the glymphatic system, which functions through CSF circulation. CSF is secreted by the epithelial cells of the choroid plexus (CP). It flows from the lateral ventricles into the third ventricle, then through the fourth ventricle, and subsequently enters the subarachnoid space surrounding the brain and spinal cord. Finally, CSF is absorbed through the arachnoid granulations into the superior sagittal sinus, where it returns to the systemic circulation [6]. The continuous movement of CSF through this pathway is crucial for removing waste from the brain [7, 8]. Recent studies underscore the vital role of normal CSF circulation in supporting cognitive function by removing neurotoxic substances, especially in ESRD patients [9–11]. For example, a study using magnetic resonance imaging (MRI) found that ESRD patients with diabetes exhibited lower CSF circulation function compared to controls, which correlated with higher neurotoxic substance levels and lower cognitive scores. However, it remains unclear whether reduced CSF circulation function in ESRD patients is associated with neurological impairment, thereby contributing to the development of MCI.

Emerging evidence suggests a cascade of events linking ESRD to neurological dysfunction, starting from the accumulation of uremic toxins and chronic inflammation [12]. These pathological processes damage cerebrovascular endothelium, disrupt the integrity of the blood–brain barrier (BBB), and impair neuronal function [13]. Astrocytic injury, oxidative stress, and abnormal aquaporin-4 (AQP4) expression may result in CSF circulation dysfunction, reducing waste clearance efficiency and promoting the accumulation of neurotoxins, which further compromise neuronal activity [14, 15]. Additionally, ESRD-associated anemia and erythropoietin deficiency may exacerbate cerebral hypoxia, amplifying CSF circulation dysfunction and its deleterious effects on the neurovascular unit (NVU), ultimately disrupting NVC [16, 17]. These changes may collectively contribute to the onset and progression of MCI. Therefore, this study hypothesizes that CSF circulation dysfunction leads to impaired NVC, thereby promoting the development of MCI in ESRD patients.

To investigate these mechanisms, non-invasive neuroimaging techniques have been increasingly applied. MRI offers a non-invasive approach to assess various aspects of CSF circulation, such as CSF production—reflected by choroid plexus volume (CPV), and interstitial fluid outflow through the perivascular spaces—quantified by the diffusion tensor imaging along the perivascular space (DTI-ALPS) index [18, 19]. In addition to the CPV and DTI-ALPS index, the perivascular space volume fraction (PVSVF) has been proposed as a structural marker reflecting glymphatic function [20]. Enlarged PVS may indicate impaired CSF influx or reduced clearance and has been associated with cognitive decline in neurodegenerative diseases, such as Alzheimer’s and Parkinson’s disease [21, 22]. Thus, in this study, we additionally evaluated PVSVF to provide complementary information on the integrity of the glymphatic system in ESRD-MCI. Furthermore, by combining resting-state functional magnetic resonance imaging (rs-fMRI) and arterial spin labeling (ASL) imaging, we can derive blood oxygen level dependent (BOLD) signals and cerebral blood flow (CBF) maps, which allow for the calculation of NVC, enabling the assessment of the coupling between brain function and blood flow perfusion in patients [23].

In this study, we aimed to explore the relationship between CSF circulation dysfunction and NVC disruption in ESRD-MCI. To evaluate the CSF circulation pathway, we measured CPV, calculated the DTI-ALPS index, and additionally assessed PVSVF. NVC was assessed using CBF and BOLD signal-derived maps and coupling metrics such as CBF-amplitude of low-frequency fluctuation (ALFF), CBF-fractional amplitude of low-frequency fluctuation (fALFF), and CBF-regional homogeneity (ReHo) coupling. Our objectives were as follows: (1) to examine whether CSF circulation dysfunction correlates with NVC alterations in ESRD-MCI and (2) to investigate whether disruptions in the CSF circulation pathway contribute to the development of MCI through NVC impairment.

## Methods

### Study protocol approvals, registration, and patient consents

This study was approved by the Ethics Committee of the First Affiliated Hospital of Xi’an Jiaotong University and registered in the Clinical Trial Registry (NCT03961724). Informed consent was obtained from all participants prior to enrollment, and the study procedures adhered to the ethical standards outlined in the Declaration of Helsinki.

### Sample size

A post hoc power analysis was conducted using G*Power (version 3.1.9.7) to evaluate the statistical power of our between-group comparisons [24]. Based on an estimated medium effect size (Cohen’s *d* = 0.5), a significance level of *α* = 0.05, and sample sizes of 68 and 65 in the ESRD and HCs groups, respectively, the achieved Power exceeded 0.80, indicating sufficient power to detect meaningful differences between groups.

### Participants

All ESRD patients were recruited from the First Affiliated Hospital of Xi’an Jiaotong University from July 2019 to December 2024. The inclusion criteria were as follows: (1) aged 18–60 years, (2) estimated glomerular filtration rate (eGFR) < 15 mL/min/1.73 m^2^, (3) Montreal cognitive assessment (MoCA) scores ≥ 18 and < 26, and (4) written informed consent. The exclusion criteria were as follows: (1) age below 18 years; (2) psychiatric or neurodegenerative disorders; (3) type I or II diabetes mellitus; (4) alcohol or drug abuse history; (5) brain lesions such as hemorrhage, stroke, tumor, encephalomalacia, or head trauma, identified through medical history or MRI scans; (6) significant visual or auditory impairments (e.g., blurred vision, hearing loss) that hinder neuropsychological assessment; and (7) claustrophobia.

We also recruited healthy controls (HCs) from the local area via poster advertisements. The exclusion criteria were consistent with those applied to the patient group.

### Demographic and cognition assessment

All participants completed a pre-enrollment questionnaire covering gender, age, and years of education, along with cognitive assessments, including the MoCA, trail making test part A (TMT-A), and auditory verbal learning test-Huashan version (AVLT-H). The MoCA is a sensitive and specific tool for MCI screening, with scores between 18 and 25 (inclusive) classified as MCI [25]. It evaluates multiple cognitive domains, including visuospatial/executive function (5 points), naming (3 points), attention (6 points), language (6 points), abstraction (2 points), delayed recall (5 points), and orientation (6 points), with a total score of 30 points. The TMT-A assesses processing speed, attention, and cognitive flexibility [26]. Participants sequentially connect 25 numbered circles, with completion time (seconds) reflecting cognitive processing speed and executive function. Longer durations indicate slower cognition. TMT-A is a widely used neuropsychological tool for detecting cognitive decline. The AVLT-H assesses verbal memory, learning ability, and delayed recall [27]. Participants memorize and recall a 12-word list over five trials, followed by delayed recall and recognition tasks. Performance is evaluated based on total immediate recall, short-term delay recall score, long-term delay recall score, and recognition accuracy. The AVLT-H is a reliable tool for assessing episodic memory and cognitive decline.

### Image acquisition

All MRI scans of the ESRD and HCs groups were acquired using a 3.0 T GE Discovery MR750 W scanner equipped with a standard head coil. The imaging protocol included three-dimensional brain volume imaging (3D-BRAVO), DTI, rs-fMRI, and 3D pseudo continuous arterial spin labelling (3D pCASL). The sequence parameters were as follows: 3D-BRAVO: repetition time (TR) = 8.5 ms, echo time (TE) = 3.2 ms, flip angle = 8°, slice thickness = 1 mm, field of view = 25.6 × 25.6 cm^2^, no gap, voxel size = 1 × 1 × 1 mm^3^, matrix = 256 × 256 mm^2^; DTI: maximum *b*-value = 1000 s/mm^2^, 30 non-collinear directions with five acquisitions without diffusion weighing (*b* = 0 s/mm^2^), TR = 9400 ms, TE = 84 ms, slice thickness = 4 mm, no gap, field of view = 24 × 24 cm^2^, voxel size = 2 × 2 × 4 mm^3^, matrix = 120 × 120 mm^2^, slices = 36; rs-fMRI: TR = 2000 ms, TE = 30 ms, flip angle = 90°, field of view = 25.6 × 25.6 cm^2^, number of volumes = 210, voxel size = 4 × 4 × 4 mm^3^, matrix = 64 × 64 mm^2^, slices = 40; 3D pCASL: TR = 4812 ms, TE = 9.8 ms, post labelling time = 2025 ms, spacing between slices = 4 mm, number of slices = 36, matrix = 128 × 128 mm^2^, flip angle = 155°.

### Image preprocessing

#### DTI images

Preprocessing of DTI data was conducted using FSL and MRtrix3 commands. The DTI images first underwent Marchenko-Pastur Principal Component (MP-PCA) denoising via “dwidenoise” followed by Gibbs unringing artifact correction using “mrdegibbs.” Subsequent distortion correction was performed in two stages: (1) eddy current compensation, (2) motion realignment through FSL’s “eddy” tool. Diffusion tensor modeling was computed using “dtifit,” generating fractional anisotropy (FA) and *x*-, *y*-, and *z*-axis diffusivity maps. Individual FA maps were spatially normalized to the JHU-ICBM-FA template using affine registration with FSL’s “flirt,” with the derived transformation matrices subsequently applied to all diffusivity maps. The superior corona radiata (SCR, projection fibers) and superior longitudinal fasciculus (SLF, association fibers) were automatically segmented at the lateral ventricle body level using the JHU-ICBM-DTI-81 atlas labels.

#### Rs-fMRI images

Data preprocessing was conducted using Statistical Parametric Mapping 12 (SPM12) and the Data Processing Assistant for Resting-State fMRI (DPARSF, http://www.restfmri.net/forum/DPARSF) [28]. First, the initial 10 functional volumes were discarded. Next, the remaining images underwent temporal correction for slice timing and head motion (24 motion-related regressors) using a least-squares approach [29]. The realigned images were then spatially normalized to the Montreal Neurological Institute (MNI) template. The normalized functional images were transformed to *Z* values, smoothed with a 4 mm full-width at half maximum (FWHM) Gaussian kernel, and bandpass filtered (0.01–0.1 Hz) to retain low-frequency fluctuations.

#### 3D pCASL images

All 3D pCASL image data were transferred to a GE Advantage Workstation (AW_46), where CBF maps were generated using the built-in 3D pCASL post-processing tool in the FUNCTOOL platform. Raw DICOM images were converted to NIFTI format using DCM2NII (available at https://people.cas.sc.edu/rorden/mricron/dcm2nii.html). Subsequent preprocessing was conducted in MATLAB (R2023a, MathWorks, Natick, MA, USA) using SPM12, University College London, available at www.fil.ion.ucl.ac.uk/spm/software/spm12/ and Data Processing and Analysis for Brain Imaging (DPABI) [30]. High-resolution T1 and CBF maps were co-registered, normalized to a standard space, and smoothed with a 4 mm FWHM Gaussian kernel.

### Cerebrospinal fluid function analysis

#### Choroid plexus volume measurement

**Fig. 1:**
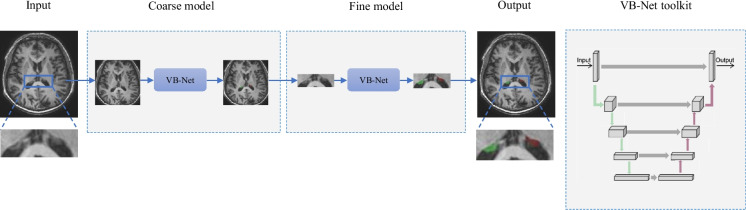
The coarse-to-fine framework of the VB-Net model for choroid plexus segmentation. The VB-Net comprised one input block, four down blocks, three up blocks, and one output block. At a low image resolution, the general positions of the choroid plexus could initially be located. Then, the edges of the choroid plexus were finely segmented at a relatively high image resolution

In the 3D-BRAVO, white matter, gray matter, and cerebrospinal fluid were automatically segmented based on the DK3 atlas. Subsequently, the entire brain was automatically partitioned into 109 subregions according to the DK109 atlas, from which the choroid plexus region was extracted. The platform employs a deep learning model called the V-shaped bottleneck network (VB-Net) [31] to train the aforementioned segmentation models. This architecture was selected for its integration of three crucial elements: an efficient encoder-decoder framework for feature embedding, residual connections for information flow, and bottleneck layers for model compression (Fig. 1). We calculated choroid plexus volumes for all participants, with subsequent normalization by their total intracranial volume (TIV).

#### Perivascular space volume fraction quantification

T1-weighted structural MRI data were processed using the Computational Anatomy Toolbox (CAT12, http://www.neuro.uni-jena.de/cat) implemented in SPM12. Images were spatially normalized to the DARTEL IXI555 MNI152 template with a voxel size of 1.5 mm. Subsequently, adaptive denoising was performed using a non-local means (NL-Means) algorithm based on the Rician noise model [32] to enhance image quality and improve the visibility and quantitative assessment of brain structures. Finally, PVSVF were segmented using a Frangi filter, which enhances tubular structures in vascular-like images based on their morphology [33].

#### DTI analysis along the perivascular space calculation

We applied the DTI-ALPS index method introduced by Taoka et al. [34]. Using ITK-SNAP software, regions of interest (ROIs) were defined as four spheres with a diameter of 5 mm in the areas of bilateral SCR and SLF, applied to all subjects’ diffusivity maps, and independently evaluated by a neuroimaging specialist. The diffusivity values in these ROIs were designated as *D*_xproj_, *D*_yproj_, *D*_zproj_, *D*_xassoc_, *D*_yassoc_, and *D*_zassoc_, allowing for the calculation of the DTI-ALPS index as follows. Subsequently, the mean DTI-ALPS index of both hemispheres was calculated.

$$\mathrm{DTI}-\text{ALPS index}= \frac{\mathrm{mean}\left({D}_{\mathrm{xproj}}, {D}_{\mathrm{xassoc}}\right)}{\mathrm{mean}\left({D}_{\mathrm{yproj}}, {D}_{\mathrm{zassoc}}\right)}$$

### BOLD FMRI data analysis

ALFF, fALFF, and ReHo maps were computed using DPARSF [28]. For ALFF and fALFF, the fast Fourier transform (FFT) was applied to decompose the time series into the frequency domain. ALFF was calculated as the mean square root of the Power spectrum within the 0.01–0.08 Hz range. fALFF was obtained as the ratio of power within this frequency band to the total power across all frequencies, reducing the influence of nonspecific signals.

For the ReHo map, band-pass filtering (0.01–0.08 Hz) was applied to the normalized images. Kendall’s coefficient of concordance (KCC) was computed to quantify ReHo by assessing the synchronization between each voxel and its neighboring voxels [35]. Finally, spatial smoothing was performed using a 4 mm FWHM Gaussian kernel.

To reduce inter-subject variability, voxel-wise ALFF, fALFF, and ReHo values were standardized by converting them into *Z*-scores. Specifically, each voxel’s ALFF and ReHo values were divided by the global mean and standard deviation across the brain, resulting in normalized *Z*-maps.

### Whole gray matter comparison of NVC metrics

To quantify NVC, whole gray matter correlations were computed between neuronal activity metrics—specifically the averaged ALFF, fALFF, and ReHo maps—and cerebral perfusion, represented by the averaged CBF maps. To ensure normality and improve comparability across subjects, the correlation coefficients were further transformed using Fisher’s *Z*-transformation. For each individual, three neurovascular coupling patterns (CBF-ALFF, CBF-fALFF, and CBF-ReHo) were assessed at the whole GM level.

### Regional comparison of NVC metrics

The Human Brainnetome Atlas (http://atlas.brainnetome.org) was used to parcellate the cerebrum into 246 regions, providing a fine-grained, cross-validated framework incorporating both anatomical and functional connectivity [36]. Regional neurovascular coupling was assessed by computing the correlation coefficients between neuronal activity metrics and cerebral perfusion across each brain region, yielding three region-based neurovascular patterns (CBF-ALFF, CBF-fALFF, and CBF-ReHo).

### Statistical analysis

#### Between-group difference of demographic and cognitive variables

All analyses were conducted using SPSS 26.0 (IBM, Armonk, NY, USA). Independent sample *t*-tests were used to compare age, years of education, and cognitive variables (MoCA, IR, SR-S, LR-S, REC, TMT-A) between groups, while sex differences were assessed with the Chi-square test. To control for potential confounders, multiple linear regression was performed to adjust for sex, age, and years of education when analyzing group differences in cognitive variables. *P* < 0.05 was considered statistically significant.

#### Between-group comparison of CPV, DTI-ALPS index, PVSVF, and NVC in ESRD

Three NVC patterns (CBF-ALFF, CBF-fALFF, and CBF-ReHo coupling) were compared between the ESRD and HCs groups at the Human Brainnetome Atlas level using independent sample *t*-tests. Additionally, CPV, DTI-ALPS index, and PVSVF were analyzed between groups using the same statistical approach. Sex, age, and years of education were included as covariates to control for confounding effects. False discovery rate (FDR) correction was applied to adjust for multiple comparisons. *P* < 0.05 was considered statistically significant.

#### Relationships between neuroimaging metrics and cognitive variables

Partial correlation analysis was conducted to assess the relationships between neuroimaging metrics and cognitive scores. Age, sex, and years of education were included as demographic covariates. To further account for individual variability in renal function severity, serum creatinine levels were also regressed out in the partial correlation analyses conducted within the ESRD group. FDR correction was applied to adjust for multiple comparisons. *P* < 0.05 was considered statistically significant.

To examine whether CSF circulation function contributes to cognitive impairment by influencing NVC, mediation analysis was performed. A simple mediation model (Model 4, PROCESS in SPSS) was used, incorporating CSF circulation function metrics as the predictor, NVC metrics as the mediator, and cognitive scores as the outcome variable. This approach, widely used due to its minimal assumptions and clear interpretability, assessed indirect effects through 10,000 bootstrap samples, estimating *β* values and 95% confidence intervals (CIs). An indirect effect was considered significant if the 95% CI did not include zero. As in the correlation analysis, age, sex, and years of education were included as covariates, and serum creatinine was additionally regressed to minimize confounding by renal dysfunction.

## Results

### Demographic and clinical characteristics

**Fig. 2:**
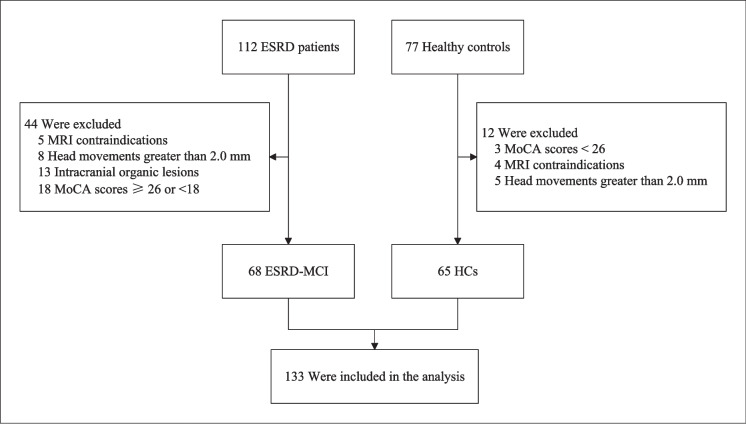
Strategy for participant enrollment and exclusion

This study recruited 189 participants from the First Affiliated Hospital of Xi’an Jiaotong University. A total of 56 participants were excluded due to MRI contraindications, excessive head motion, intracranial organic lesions, or invalid MoCA scores. The final sample consisted of 133 participants (68 ESRD patients with MCI and 65 HCs) (Fig. 2).

For age, sex, and years of education, there were statistical differences (*P* < 0.05) between the ESRD (41 females) and HCs (27 females) groups. For the MoCA, TMT-A, IR, SR-S, and LR-S scores, statistically significant differences (*P* < 0.001) were found between the ESRD and HCs groups. However, no significant difference was observed in REC scores (*P* > 0.05) between the groups (Table 1).

**Table 1:** Demographic and clinical variables

Variables	ESRD (*N* = 68)	HCs (*N* = 65)	Statistics	*P* values
Age, mean (SD)	41.04 (11.68)	35.22 (13.31)	2.680^$^	0.008
Female, *n* (%)	41 (60.3%)	27 (41.5%)	4.679^#^	0.031
Years of education, mean (SD)	10.35 (2.85)	12.46 (2.12)	− 4.856^$^	< 0.001
MoCA, mean (SD)	22.51 (2.18)	28.63 (1.23)	− 12.930^δ^	< 0.001
TMT-A, s, mean (SD)	68.67 (38.01)	37.74 (10.34)	4.786^δ^	< 0.001
IR score, mean (SD)	22.37 (6.14)	28.35 (3.44)	− 4.535^δ^	< 0.001
SR-S score, mean (SD)	8.26 (2.58)	10.40 (1.18)	− 3.738^δ^	< 0.001
LR-S score, mean (SD)	7.78 (2.49)	10.34 (1.25)	− 4.990^δ^	< 0.001
REC score, mean (SD)	15.82 (5.59)	15.58 (5.53)	− 1.417^δ^	0.159
Serum creatinine (μmol/L)	815.96 (245.13)	-	-	-

^#^Chi-square test; ^$^independent samples test; ^δ^independent samples test on residuals from regression with sex, age, and years of education as covariates. *ESRD* end-stage renal disease, *HCs* healthy controls, *MoCA* Montreal cognitive assessment, *TMT-A* trail making test part A, *IR* immediate recall, *SR-S* short-term delay recall score, *LR-S* long-term delay recall score, *REC* recognition, *SD* standard deviation

### Between-group comparison of CPV, DTI-ALPS index, and PVSVF

**Fig. 3:**
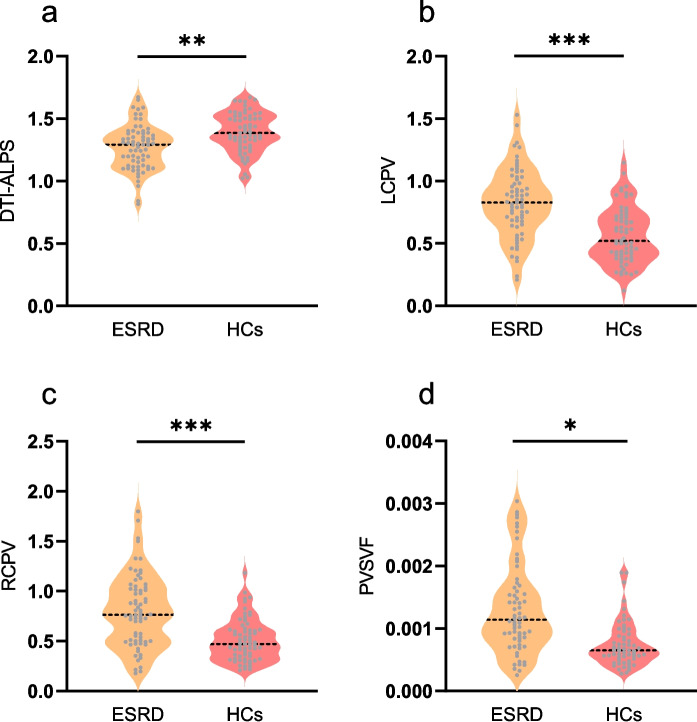
Group comparisons of cerebrospinal fluid circulation function metrics (**a** DTI-ALPS index, **b** LCPV, **c** RCPV, **d** PVSVF) in ESRD and HCs groups. *P < 0.05; **P < 0.01; ***P < 0.001. ESRD, end-stage renal disease; HCs, healthy controls; DTI-ALPS, diffusion tensor imaging along the perivascular space; LCPV, left choroid plexus volume; RCPV, right choroid plexus volume; PVSVF, perivascular space volume fraction

ESRD patients exhibited significantly higher CPV (*P* < 0.001) and PVSVF (*P* < 0.05), as well as a significantly lower DTI-ALPS index (*P* < 0.01) compared to HCs (Fig. 3).

### Between-group comparison of whole gray matter NVC metrics

**Fig. 4:**
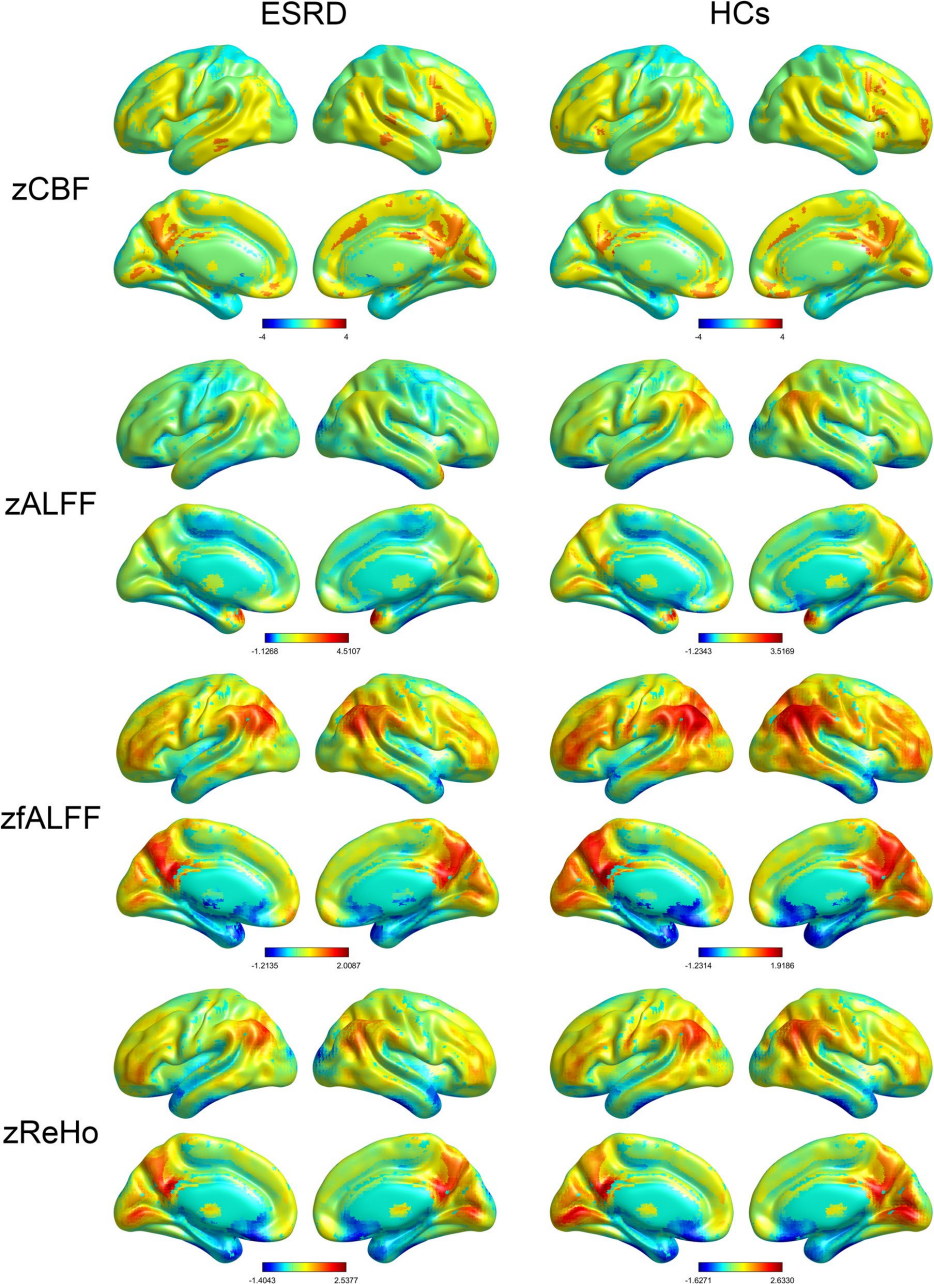
Spatial distribution of averaged CBF, ALFF, fALFF, and ReHo *Z*-maps. Maps represent group-level averages across all subjects within each group. ESRD, end-stage renal disease; HCs, healthy controls; CBF, cerebral blood flow; ALFF, amplitude of low frequency fluctuation; fALFF, fractional amplitude of low frequency fluctuation; ReHo, regional homogeneity

**Fig. 5:**
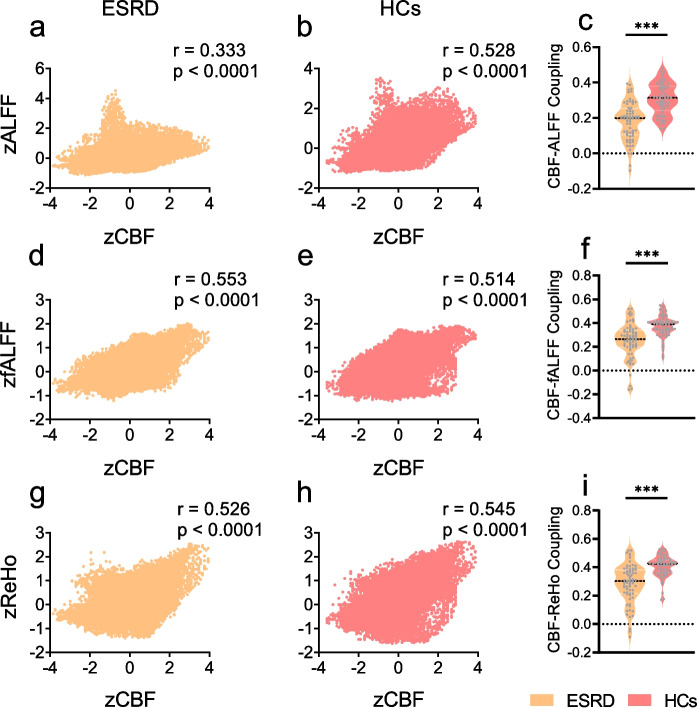
CBF-ALFF (**a**–**c**), CBF-fALFF (**d**–**f**), and CBF-ReHo coupling (**g**–**i**) at the global level in ESRD and HCs groups. ***P < 0.001. ESRD, end-stage renal disease; HCs, healthy controls; CBF, cerebral blood flow; ALFF, amplitude of low frequency fluctuation; fALFF, fractional amplitude of low frequency fluctuation; ReHo, regional homogeneity

Average global CBF, ALFF, fALFF, and ReHo *Z*-score maps for both groups are presented in Fig. 4. Visual inspection revealed distinct distribution patterns between ESRD patients and HCs, suggesting potential differences in NVC patterns. Significant across-voxel correlations between CBF and neuronal activity (ALFF, fALFF, and ReHo) were observed in both ESRD patients and HCs (all *P* < 0.001, Fig. 5). At the whole gray matter level, NVC was assessed using three metrics: CBF-ALFF, CBF-fALFF, and CBF-ReHo coupling coefficients. ESRD patients exhibited significantly lower global NVC coupling across all three measures compared to HCs (all *P* < 0.001, Fig. 5).

### Between-group comparison of regional NVC metrics

**Fig. 6:**
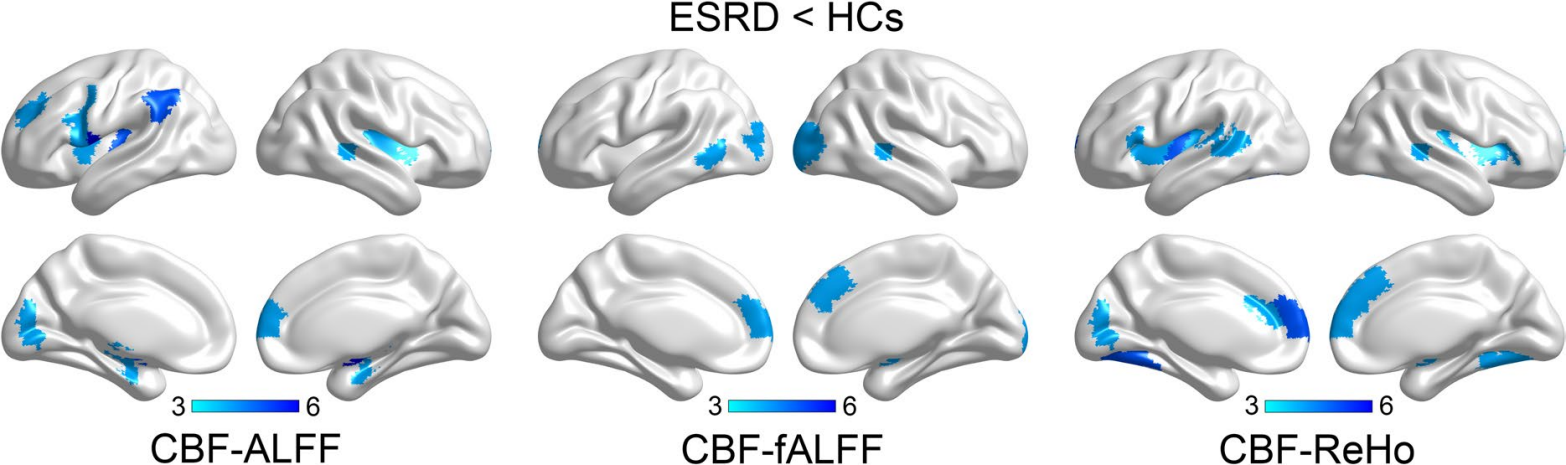
*T*-value maps of brain regions with significantly abnormal NVC, false discovery rate corrected. NVC, neurovascular coupling; CBF, cerebral blood flow; ALFF, amplitude of low frequency fluctuation; fALFF, fractional amplitude of low frequency fluctuation; ReHo, regional homogeneity

ESRD patients exhibited significantly lower NVC coupling coefficients compared to HCs across multiple brain regions (*P*_FDR_ < 0.05, Fig. 6, Table S1). CBF-ALFF coupling was significantly lower in 24 regions, involving the frontal gyrus, precentral gyrus, parietal gyrus, insular gyrus, occipital cortex, basal ganglia, hippocampus, and thalamus. CBF-fALFF coupling was significantly lower in 10 regions, involving the frontal gyrus, temporal gyrus, occipital gyrus, amygdala, and caudate. CBF-ReHo coupling was significantly lower in 24 regions, involving the frontal gyrus, temporal gyrus, fusiform gyrus, insular gyrus, cingulate gyrus, occipital cortex, amygdala, and caudate.

### Relationships between CPV, DTI-ALPS index, PVSVF, and NVC

**Fig. 7:**
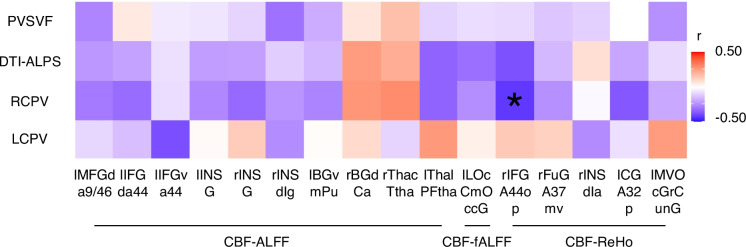
Relationships between NVC and cerebrospinal fluid circulation function metrics in ESRD, after adjustment for sex, age, years of education, and serum creatinine levels. Results are corrected using the false discovery rate. lMFGda9/46, left middle frontal gyrus dorsal area 9/46; lIFGda44, left inferior frontal gyrus dorsal area 44; lIFGva44, left inferior frontal gyrus ventral area 44; lINSG, left hypergranular insula; rINSG, right hypergranular insula; rINSdIg, right dorsal granular insula; lBGvmPu, left basal ganglia ventromedial putamen; rBGdCa, right basal ganglia dorsal caudate; rThacTtha, right caudal temporal thalamus; lThalPFtha, left lateral pre-frontal thalamus; lLOcCmOccG, left lateral occipital cortex middle occipital gyrus; rIFGA44op, right inferior frontal gyrus opercular area 44; rFuGA37mv, right fusiform gyrus medioventral area 37; rINSdIa, right dorsal agranular insula; lCGA32p, left cingulate gyrus pregenual area 32; lMVOcGrCunG, left medioventral occipital cortex rostral cuneus gyrus; CBF, cerebral blood flow; ALFF, amplitude of low frequency fluctuation; fALFF, fractional amplitude of low frequency fluctuation; ReHo, regional homogeneity; PVSVF, perivascular space volume fraction; DTI-ALPS, diffusion tensor imaging along the perivascular space; RCPV, right choroid plexus volume; LCPV, left choroid plexus volume

After controlling for sex, age, years of education, and serum creatinine levels, regional NVC metrics remained significantly associated with CPV. Specifically, CBF-ReHo coupling of right inferior frontal gyrus opercular area 44 was negatively correlated with RCPV (*P*_FDR_ < 0.05) (Fig. 7).

### Partial correlation and mediation analysis between CPV, DTI-ALPS index, PVSVF, and NVC

**Fig. 8:**
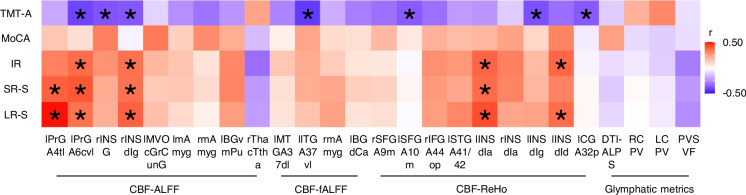
Relationships between neuroimaging metrics and clinical variables in ESRD, after adjustment for sex, age, years of education, and serum creatinine levels. Results are corrected using the false discovery rate. TMT-A, trail making test part A; MoCA, Montreal cognitive assessment; IR, immediate recall; SR-S, short-term delay recall score; LR-S, long-term delay recall score; lPrGA4tl, left precentral gyrus area 4 (tongue and larynx region); lPrGA6cvl, left precentral gyrus caudal ventrolateral area 6; rINSG, right hypergranular insula; rINSdIg, right dorsal granular insular; lMVOcGrCunG, left medioventral occipital cortex rostral cuneus gyrus; lmAmyg, left medial amygdala; rmAmyg, right medial amygdala; lBGvmPu, left basal ganglia ventromedial putamen; rThacTtha, right caudal temporal thalamus; lMTGA37dl, left middle temporal gyrus dorsolateral area 37; lITGA37vl, left inferior temporal gyrus ventrolateral area 37; rmAmyg, right medial amygdala; lBGdCa, left basal ganglia dorsal caudate; rSFGA9m, right superior frontal gyrus medial area 9; lSFGA10m, left superior frontal gyrus medial area 10; lIFGA44op, left inferior frontal gyrus opercular area 44; lSTGA41/42, left superior temporal gyrus area 41/42; lINSdIa, left dorsal agranular insula; rINSdIa, right dorsal agranular insula; lINSdIg, left dorsal granular insula; lINSdId, left dorsal dysgranular insula; lCGA32p, left cingulate gyrus pregenual area 32; CBF, cerebral blood flow; ALFF, amplitude of low frequency fluctuation; fALFF, fractional amplitude of low frequency fluctuation; ReHo, regional homogeneity; DTI-ALPS, diffusion tensor imaging along the perivascular space; RCPV, right choroid plexus volume; LCPV, left choroid plexus volume; PVSVF, perivascular space volume fraction

**Fig. 9:**
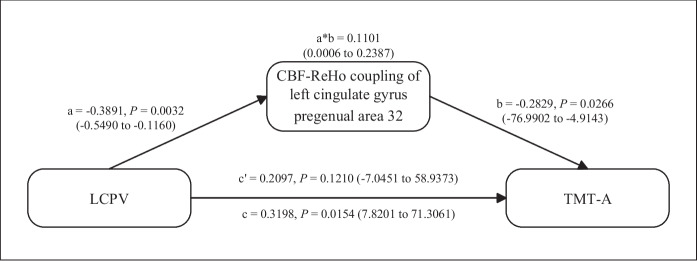
Mediation effects of CBF-ReHo coupling on the association between elevated LCPV and TMT-A scores, adjusted for sex, age, years of education, and serum creatinine levels. LCPV, left choroid plexus volume; CBF, cerebral blood flow; ReHo, regional homogeneity; TMT-A, trail making test part A

After controlling for sex, age, years of education, and serum creatinine levels, partial correlation analysis revealed significant associations between regional NVC metrics and cognitive scores. These associations remained significant following FDR correction (Fig. 8). After controlling for sex, age, years of education, and serum creatinine levels, mediation analysis further indicated that CBF-ReHo coupling in the left cingulate gyrus pregenual area 32 served as a significant mediator in the relationship between LCPV and cognitive impairment. Specifically, increased LCPV may contribute to cognitive decline by reducing regional CBF-ReHo coupling (*β* = 0.1101, *P* < 0.05, CIs = [0.0006 to 0.2387]) (Fig. 9).

## Discussion

### Main findings

In this study, we aimed to investigate whether CSF circulation dysfunction correlates with NVC alterations in ESRD-MCI and to explore whether disruptions in the CSF circulation pathway contribute to the development of MCI through NVC impairment. Our findings revealed that, compared to HCs, ESRD patients with MCI exhibited significantly lower DTI-ALPS index and both global and regional NVC, while CPV and PVSVF were significantly higher. Additionally, CPV showed a significant negative correlation with regional NVC in ESRD patients. Finally, CBF-ReHo coupling in the left cingulate gyrus was identified as a potential mediator linking increased LCPV and cognitive scores. These results suggest that abnormal CSF circulation contributes to the development of MCI in ESRD patients by affecting NVC patterns.

### CSF circulation dysfunction in ESRD with MCI

Normal CSF circulation in the brain ensures the glymphatic system’s ability to clear metabolic waste. The CP plays a crucial role in the CSF production process and may serve as a driving force for CSF circulation [37, 38]. Research has shown that a larger CP volume is associated with lower CSF turnover in neurodegenerative diseases [37, 39] and ESRD patients with cognitive impairment [11, 40]. The DTI-ALPS index can reflect the process of CSF outflow from the brain. Recent studies have shown that the DTI-ALPS index is significantly reduced in Alzheimer’s disease patients compared to healthy controls and is associated with cognitive decline [41, 42]. In ESRD patients, a lower DTI-ALPS index was associated with worse cognitive performance [4]. In 2024, the founder of the DTI-ALPS index proposed that multiple neuroimaging metrics should be combined to assess CSF circulation function within the glymphatic system [18]. A study by Hu et al. further confirmed the reliability of combining the CPV and DTI-ALPS index to assess CSF circulation function [43]. Our findings demonstrated lower DTI-ALPS index and higher CPV in ESRD patients with MCI compared to HCs. Although an initial association was observed between LCPV and cognitive scores after adjusting for sex, age, years of education, and serum creatinine levels, the correlation did not survive correction for multiple comparisons and should therefore be interpreted with caution. Nevertheless, given the central role of the CP in CSF dynamics, its potential relevance to cognitive function warranted further exploration, which was addressed in subsequent mediation analyses. In addition to the CPV and DTI-ALPS index, we assessed PVSVF as an auxiliary indicator of glymphatic integrity. ESRD patients with MCI showed significantly enlarged PVSVF. However, although a trend-level correlation was observed between PVSVF and cognitive scores after covariate adjustment, the association did not survive correction for multiple comparisons. This suggests that PVSVF may reflect structural alterations rather than robust functional changes. Therefore, its interpretation should be cautious, and future studies should incorporate dynamic CSF flow imaging to comprehensively assess glymphatic function. Nonetheless, the observed trends support the hypothesis that impaired CSF circulation may disrupt brain waste clearance and contribute to cognitive decline in ESRD-MCI.

### NVC dysfunction in ESRD with MCI

Recent studies have highlighted the role of NVC dysfunction in cognitive decline in ESRD patients [23, 44]. Our findings confirm these impairments, identifying specific regional NVC alterations in areas such as the insular gyrus, cingulate gyrus, frontal gyrus, temporal gyrus, and occipital cortex, and their association with cognitive impairment in ESRD-MCI, thereby extending previous research. The insular gyrus and cingulate gyrus are crucial for attention and cognitive control, with ALFF and ReHo studies showing reduced activation in these areas in patients with cognitive impairment [45, 46]. Abnormalities in NVC within the frontal gyrus and temporal gyrus have also been reported, with studies indicating cortical dysfunction and altered blood flow in ESRD patients with cognitive decline [47]. Our study further confirms this. The frontal gyrus is essential for executive functions, including decision-making, working memory, and higher-order cognitive processes [48], while the temporal gyrus, particularly the superior temporal gyrus, is involved in language comprehension and memory [49]. The occipital cortex, primarily responsible for visual processing, also supports cognitive tasks [50]. These findings suggest that NVC dysfunction in these regions may contribute to the development of MCI in ESRD, particularly in memory, attention, and executive functions, reflecting a complex network of cognitive control. Overall, our results further support NVC dysfunction as a potential pathophysiological mechanism of ESRD-MCI. In summary, this study found that both CSF circulation dysfunction and NVC occur in ESRD-MCI with possible common pathophysiological mechanisms.

### Relationship between impaired CSF circulation function and dysfunctional NVC, and cognitive scores in ESRD with MCI

Recent evidence suggests that NVC plays a role in both cerebrospinal fluid inflow and outflow, in addition to oxygen supply to neurons [51]. In this study, we found significant negative correlations between multiple regional NVC patterns and indicators of CSF circulation function. These findings align with those of Fultz and colleagues, who confirmed the inverse between brain BOLD signals and CSF flow signals during both wakefulness and sleep [52]. Additionally, a recent study demonstrated that NVC stimulation accelerates CSF drainage and metabolite removal under anesthesia [53], highlighting the promoting effect of NVC on CSF circulation function. However, in ESRD-MCI patients, there may be a unique interaction pattern between the CSF circulation and NVC. Due to kidney function loss, the accumulation of uremic toxins and chronic inflammatory factors commonly leads to microvascular damage, compromising the BBB’s structural integrity over time [54], which exacerbates neuronal toxic damage. Thus, we speculate that CSF circulation dysfunction may impair NVC, subsequently affecting cognitive performance. Mediation analysis revealed that CBF-ReHo coupling in the left cingulate gyrus pregenual area 32 mediates the relationship between increased LCPV and cognitive decline, supporting our hypothesis. Our findings highlight the intricate interplay between the CSF circulation and NVC in the context of ESRD-MCI, suggesting that CSF circulation dysfunction and compromised NVC may synergistically contribute to cognitive decline. To minimize the potential confounding effects of systemic disease severity, all within-ESRD analyses in this study were statistically adjusted for sex, age, years of education, and serum creatinine levels. The persistence of significant associations after this adjustment suggests that the observed alterations in CSF circulation and NVC are not merely consequences of biochemical derangements, but may instead reflect more extensive neurovascular and glymphatic system dysfunction in ESRD.

There are some limitations of our study to be acknowledged. First, although we adjusted for age, sex, and years of education in all statistical analyses, there were still significant between-group differences in these demographic variables. Such differences may introduce residual confounding effects that could influence both cognitive performance and neuroimaging outcomes. Therefore, the potential impact of demographic imbalance should be acknowledged, and future studies should consider matched sampling designs to reduce these biases. Second, imaging metrics such as the DTI-ALPS index, CPV, and PVSVF are indirect and may not fully reflect glymphatic function. Finally, it is also important to acknowledge several methodological limitations in interpreting the DTI-ALPS index. As noted by Taoka et al. this metric may be affected by factors such as imaging plane orientation, *b*-value selection, and head positioning during scanning. In this study, we followed a standardized DTI protocol across all participants to minimize variability. Nonetheless, subtle inter-individual anatomical differences and the static nature of DTI remain unavoidable limitations. Future work incorporating dynamic imaging techniques and automated ROI placement may further improve the reliability of glymphatic assessment.

In conclusion, this study highlights significant impairments in CSF circulation function and NVC in ESRD patients with MCI, with CPV and NVC metrics serving as potential biomarkers for cognitive decline. The findings suggest that CSF circulation dysfunction and NVC impairment may jointly contribute to cognitive deterioration in these patients, emphasizing the potential of targeting CSF circulation pathway and NVC for early detection and intervention in ESRD-MCI.

## Supplementary Information

Below is the link to the electronic supplementary material.

Supplementary file1 (DOCX 24 kb)

## Data Availability

The data are available upon reasonable request and with prior approval from the Ethics Committee of the First Affiliated Hospital of Xi’an Jiaotong University.
