# Supplementary material for: Dysfunction of cerebrospinal fluid circulation and neurovascular decoupling in end-stage renal disease patients with mild cognitive impairment
Source: GeroScience. 2025 Oct 12;48(3):4491–507. doi: 10.1007/s11357-025-01901-5 (PMC13355962; doi:10.1007/s11357-025-01901-5)
Supplement: Supplementary file 1 — Supplementary file1 (DOCX 24 kb) [file 11357_2025_1901_MOESM1_ESM.docx]

Table S1 Between-group comparison of NVC of ESRD patients and HCs at brain regional level

| Brain regions | MNI coordinates | | | *T* values | *P_FDR_* values |
| --- | --- | --- | --- | --- | --- |
|  | X | Y | Z |  |  |
| ESRD < HCs |  |  |  |  |  |
| CBF-ALFF |  |  |  |  |  |
| Right superior frontal gyrus medial area 10 | 8 | 58 | 13 | 4.170 | < 0.001 |
| Left middle frontal gyrus dorsal area 9/46 | -27 | 43 | 31 | 4.090 | < 0.001 |
| Left inferior frontal gyrus dorsal area 44 | -46 | 13 | 24 | 3.612 | < 0.001 |
| Left inferior frontal gyrus ventral area 44 | -52 | 13 | 6 | 3.838 | < 0.001 |
| Left precentral gyrus area 4 | -52 | 0 | 8 | 5.509 | <0.001 |
| Left precentral gyrus caudal ventrolateral area 6 | -49 | 5 | 30 | 3.964 | < 0.001 |
| Right rostroposterior superior temporal sulcus | 53 | -37 | 3 | 3.856 | < 0.001 |
| Left inferior parietal lobule caudal area 40 | -56 | -49 | 38 | 4.560 | < 0.001 |
| Left insular gyrus hypergranular insula | -36 | -20 | 10 | 4.538 | < 0.001 |
| Right insular gyrus hypergranular insula | 37 | -18 | 8 | 4.262 | < 0.001 |
| Left insular gyrus dorsal granular insular | -38 | -8 | 8 | 3.498 | < 0.001 |
| Right insular gyrus dorsal granular insular | 39 | -7 | 8 | 4.335 | < 0.001 |
| Left insular gyrus dorsal dysgranular insula | -38 | 5 | 5 | 4.280 | < 0.001 |
| Right insular gyrus dorsal dysgranular insula | 38 | 5 | 5 | 3.598 | < 0.001 |
| Left medioventral occipital cortex rostral cuneus gyrus | -5 | -81 | 10 | 4.098 | < 0.001 |
| Left medial amygdala | -19 | -2 | -20 | 5.178 | < 0.001 |
| Right medial amygdala | 19 | -2 | -19 | 5.618 | < 0.001 |
| Left rostral hippocampus | -22 | -14 | -19 | 3.767 | < 0.001 |
| Right rostral hippocampus | 22 | -12 | -20 | 3.765 | < 0.001 |
| Right ventral caudate | 15 | 14 | -2 | 4.101 | < 0.001 |
| Left ventromedial putamen | -23 | 7 | -4 | 3.949 | < 0.001 |
| Left dorsal caudate | -14 | 2 | 16 | 5.059 | < 0.001 |
| Right dorsal caudate | 14 | 5 | 14 | 4.716 | < 0.001 |
| Left lateral pre-frontal thalamus | -11 | -14 | 2 | 3.973 | < 0.001 |
| CBF-fALFF |  |  |  |  |  |
| Right superior frontal gyrus medial area 9 | 6 | 38 | 35 | 3.580 | < 0.001 |
| Left superior frontal gyrus medial area 10 | -8 | 56 | 15 | 3.625 | < 0.001 |
| Left middle temporal gyrus dorsolateral area 37 | -59 | -58 | 4 | 3.722 | < 0.001 |
| Left inferior temporal gyrus ventrolateral area 37 | -55 | -60 | -6 | 4.180 | < 0.001 |
| Right rostroposterior superior temporal sulcus | 53 | -37 | 3 | 3.854 | < 0.001 |
| Left middle occipital gyrus | -31 | -89 | 11 | 3.591 | < 0.001 |
| Right middle occipital gyrus | 34 | -86 | 11 | 4.418 | < 0.001 |
| Right occipital polar cortex | 22 | -97 | 4 | 4.300 | < 0.001 |
| Right medial amygdala | 19 | -2 | -19 | 3.735 | < 0.001 |
| Left dorsal caudate | -14 | 2 | 16 | 4.564 | < 0.001 |
| CBF-DC |  |  |  |  |  |
| Left superior frontal gyrus dorsolateral area 6 | -18 | -1 | 65 | 4.302 | < 0.001 |
| Right superior frontal gyrus medial area 10 | 8 | 58 | 13 | 3.744 | < 0.001 |
| Left precentral gyrus caudal dorsolateral area 6 | -32 | -9 | 58 | 3.586 | < 0.001 |
| Left precentral gyrus area 4 | -52 | 0 | 8 | 4.494 | < 0.001 |
| Right fusiform gyrus lateroventral area 37 | 43 | -49 | -19 | 4.096 | < 0.001 |
| Left medioventral occipital cortex rostral cuneus gyrus | -5 | -81 | 10 | 3.779 | < 0.001 |
| Right medial amygdala | 19 | -2 | -19 | 4.921 | < 0.001 |
| Left dorsal caudate | -14 | 2 | 16 | 5.257 | < 0.001 |
| Right dorsal caudate | 14 | 5 | 14 | 3.658 | < 0.001 |
| CBF-ReHo |  |  |  |  |  |
| Right superior frontal gyrus medial area 9 | 6 | 38 | 35 | 3.507 | < 0.001 |
| Left superior frontal gyrus medial area 10 | -8 | 56 | 15 | 4.630 | < 0.001 |
| Right superior frontal gyrus medial area 10 | 8 | 58 | 13 | 4.085 | < 0.001 |
| Left inferior frontal gyrus caudal area 45 | -53 | 23 | 11 | 3.592 | < 0.001 |
| Right inferior frontal gyrus opercular area 44 | 42 | 22 | 3 | 3.567 | < 0.001 |
| Left superior temporal gyrus area 41/42 | -54 | -32 | 12 | 3.585 | < 0.001 |
| Left fusiform gyrus medioventral area 37 | -31 | -64 | -14 | 4.622 | < 0.001 |
| Right fusiform gyrus medioventral area 37 | 31 | -62 | -14 | 3.899 | < 0.001 |
| Left rostroposterior superior temporal sulcus | -54 | -40 | 4 | 3.753 | < 0.001 |
| Right rostroposterior superior temporal sulcus | 53 | -37 | 3 | 4.104 | < 0.001 |
| Left caudoposterior superior temporal sulcus | -52 | -50 | 11 | 3.674 | < 0.001 |
| Left insular gyrus hypergranular insula | -36 | -20 | 10 | 3.823 | < 0.001 |
| Right insular gyrus hypergranular insula | 37 | -18 | 8 | 3.644 | < 0.001 |
| Left insular gyrus dorsal agranular insula | -34 | 18 | 1 | 3.560 | < 0.001 |
| Right insular gyrus dorsal agranular insula | 36 | 18 | 1 | 3.666 | < 0.001 |
| Left insular gyrus dorsal granular insula | -38 | -8 | 8 | 4.876 | < 0.001 |
| Left insular gyrus dorsal dysgranular insula | -38 | 5 | 5 | 3.824 | < 0.001 |
| Right insular gyrus dorsal dysgranular insula | 38 | 5 | 5 | 3.956 | < 0.001 |
| Left cingulate gyrus pregenual area 32 | -6 | 34 | 21 | 4.419 | < 0.001 |
| Left medioventral occipital cortex caudal lingual gyrus | -11 | -82 | -11 | 3.484 | < 0.001 |
| Left medioventral occipital cortex caudal rostral cuneus gyrus | -5 | -81 | 10 | 3.563 | < 0.001 |
| Right medial amygdala | 19 | -2 | -19 | 4.320 | < 0.001 |
| Left dorsal caudate | -14 | 2 | 16 | 4.175 | < 0.001 |
| Right dorsal caudate | 14 | 5 | 14 | 4.465 | < 0.001 |

ESRD: end-stage renal disease; HCs: healthy controls; NVC: neurovascular coupling; MNI: Montreal neurological institute; CBF: cerebral blood flow; ALFF: amplitude of low frequency fluctuation; fALFF: fractional amplitude of low frequency fluctuation; DC: degree centrality; ReHo: regional homogeneity.
